# Bioinspired Honeycomb Core Design: An Experimental Study of the Role of Corner Radius, Coping and Interface

**DOI:** 10.3390/biomimetics5040059

**Published:** 2020-11-04

**Authors:** Derek Goss, Yash Mistry, Sridhar Niverty, Cameron Noe, Bharath Santhanam, Cahit Ozturk, Clint A. Penick, Christine Lee, Nikhilesh Chawla, Alex Grishin, Vikram Shyam, Dhruv Bhate

**Affiliations:** 1 3DX Research Group, The Polytechnic School, Arizona State University, Mesa, AZ 85212, USA; dlgoss@asu.edu (D.G.); ymistry@asu.edu (Y.M.); cnoe1@asu.edu (C.N.); bsantha4@asu.edu (B.S.); 2 School of Materials Engineering, Purdue University, West Lafayette, IN 47907, USA; sniverty@purdue.edu (S.N.); nikc@purdue.edu (N.C.); 3 Bee Research Lab, School of Life Sciences, Arizona State University, Mesa, AZ 85212, USA; cahit.ozturk@asu.edu; 4 Ecology, Evolution & Organismal Biology, Kennesaw State University, Kennesaw, GA 30144, USA; cpenick1@kennesaw.edu; 5 Interwoven Labs, School of Art, Arizona State University, Tempe, AZ 85281, USA; christinelee@asu.edu; 6 Phoenix Analysis & Design Technologies, Inc., Tempe, AZ 85284, USA; alex.grishin@padtinc.com; 7 NASA Glenn Research Center, Cleveland, OH 44135, USA; vikram.shyam-1@nasa.gov

**Keywords:** honeycomb, optimization, additive manufacturing, bioinspired design, biomimicry, compression, three-point bending

## Abstract

The honeybee’s comb has inspired the design of engineering honeycomb core that primarily abstract the hexagonal cell shape and exploit its mass minimizing properties to construct lightweight panels. This work explored three additional design features that are part of natural honeybee comb but have not been as well studied as design features of interest in honeycomb design: the radius at the corner of each cell, the coping at the top of the cell walls, and the interface between cell arrays. These features were first characterized in natural honeycomb using optical and X-ray techniques and then incorporated into honeycomb core design and fabricated using an additive manufacturing process. The honeycomb cores were then tested in out-of-plane compression and bending, and since all three design features added mass to the overall structure, all metrics of interest were examined per unit mass to assess performance gains despite these additions. The study concluded that the presence of an interface increases specific flexural modulus in bending, with no significant benefit in out-of-plane compression; coping radius positively impacts specific flexural strength, however, the corner radius has no significant effect in bending and actually is slightly detrimental for out-of-plane compression testing.

## 1. Introduction

The hexagonal honeycomb has been the subject of great interest and study for millennia, from Euclid in the 4th century B.C. admiring the geometry of their cells [1] to the mathematician Hales, who formally proved the “honeycomb conjecture” in a 2001 paper, showing once and for all that the hexagonal pattern was indeed the most efficient partitioning of two-dimensional space into equal areas [2]. The hexagonal cell has also inspired a wide range of engineering products, summarized in a review by Zhang et al. [3], and been the subject of a great deal of analytical modeling [4,5,6,7], as well as computational and experimental study [8,9,10].

The vast majority of prior work places emphasis on the hexagonal cell shape itself, the optimization of the key dimensional parameters associated with it, namely, the size of the individual hexagonal cell and the thickness of the cell wall, and the integration of the cellular structure into the application of interest. An example of the latter is the selection and bonding of sandwiching sheets to the honeycomb core, a critical selection for the manufacturing of honeycomb panel. This focus is appropriate, since these are the primary design variables of interest when designing cellular materials [11] and also because manufacturing processes for honeycombs have traditionally been constrained in their level of control of features. These constraints have been greatly reduced with the advent of additive manufacturing (AM) technologies, making it possible now to manufacture honeycomb structures with an unprecedented level of design freedom. It is in this context that this research seeks to make a closer examination of natural honeybee comb, focusing on geometric features of interest beyond just the familiar hexagonal cell motif. Three such geometric features, which are the focus of this work, are the corner radius, coping, and the interface, each described in more detail below. The objective of this research is to examine what role these design parameters have to play in the mechanical behavior of honeycomb cores that form the interior of honeycomb panels. All of these parameters and their benefits are of interest in the context of mass minimization—this is a driving requirement both for the honeybees, who expend significant calories producing wax and constructing these combs, as well as for honeycomb panel applications, particularly in the aerospace industry, where weight reduction is a critical area of concern.

### 1.1. Corner Radius

**Figure 1:**
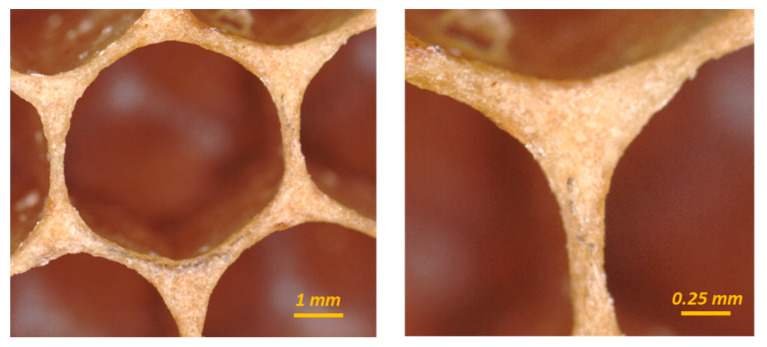
The corners of a hexagonal cell in a honeybee (*A. mellifera*) comb have clearly visible fillet radii.

The corners of a hexagonal cell in a honeybee comb have clearly visible fillet radii, as shown in Figure 1. This has been used to argue that these cells are initially circular, but “quickly transform into the familiar rounded hexagonal shape while the comb is being built” [12]. However, a significant amount of beeswax is invested in the corners of these cells that could have otherwise been allocated elsewhere. Therefore, of interest to the current study is what structural advantage, per unit mass, this corner radius provides.

### 1.2. Coping

**Figure 2:**
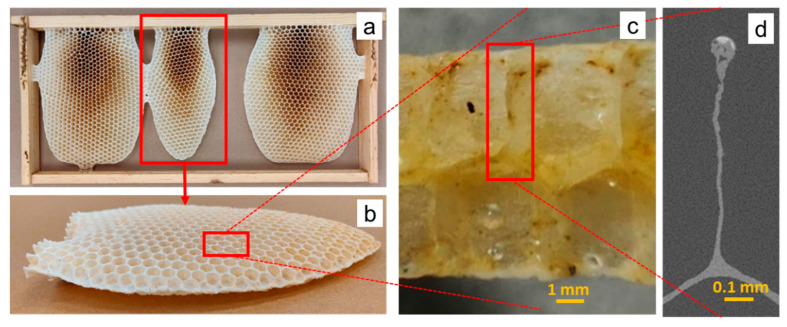
Coping in the walls of a bee’s natural honeycomb: (**a**) honeycomb made by *A. mellifera* in an open comb frame without any backing, (**b**) freshly built comb broken off, (**c**) laser-cut piece of comb, viewed side on, and (**d**) X-ray tomography slice showing that the cell wall terminates with clearly evident coping.

Just as a closer view of the two-dimensional cell reveals the presence of significant corner radii, a closer view of a section of a cell wall reveals that honeycomb cells do not have uniform thickness, as shown in Figure 2d. Although there is some expected natural variation in the thickness along the wall, there is a very clear matchstick-shaped coping at the top of the wall, where it terminates. As with the corner radii, this represents an addition of mass, suggesting some functional benefit. This coping diminishes over time, but is very evident in freshly built comb (i.e., comb that is less than a year old, prior to the emergence of the first brood from that comb), in particular [13]. Comb structure changes after the first year as the walls comprised initially exclusively of beeswax, gradually see an influx of silk fibers from the emerged brood’s cocoons, which are woven into the walls and improve its mechanical properties and also reduce its sensitivity to temperature [13,14].

### 1.3. Interface

**Figure 3:**
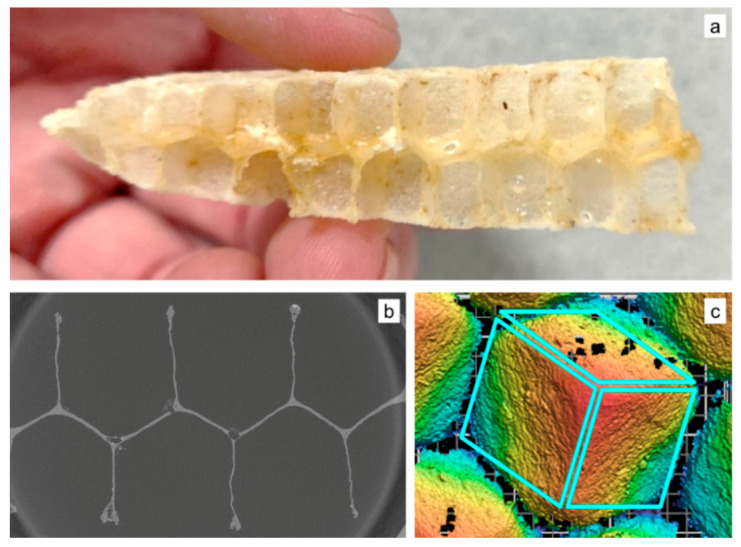
(**a**) Honeybee combs are constructed of two opposite facing arrays of cells, (**b**) which in a sectional, two-dimensional view shows a zigzag pattern and (**c**) in three dimensions has the appearance of a trihedral pyramid.

Honeybees build their comb with two arrays of unit cells (see Figure 3a) stacked such that the ends intersect each other in a zigzag pattern when viewed in two dimensions, as shown in Figure 3b. The intersection of the two arrays is what is termed as the interface for this study, since it is the surface that separates the cells into two arrays. The interface within an individual cell can be idealized by three planar rhombi, or as the top of a trihedral pyramid, shown in Figure 3c, although this has been found to be the case only for freshly built comb, with the shape gradually evolving into a spherical one as the comb gets older [15]. Although mathematicians have shown that there are shapes that improve on this intersecting pattern, they do so fractionally and involve structures of greater complexity where the effort may not justify the slight benefit [1].

Corner radius, coping, and interface are the three design features studied in this work, and subjected to an experimental study of honeycomb cores with varying parametric values of these features. The focus of this work is on examining the mechanical benefits of these geometric features, examining them per unit mass for a more appropriate comparison against baseline honeycomb core with no corner radii, coping, or interface. Two test modalities are addressed in this work: out-of-plane compression and bending.

## 2. Materials and Methods

### 2.1. Overall Approach

**Figure 4:**
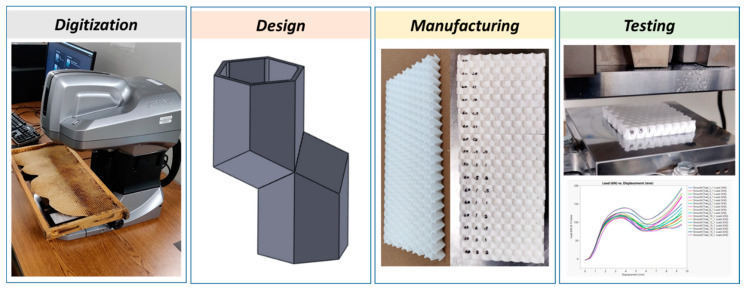
Overall approach in this work, from digitization through testing.

The approach taken in this study is demonstrated in Figure 4 and consists of four main steps. Digitization and quantification have to do with identifying features in natural honeybee comb and, where applicable, quantifying them. The intent of this step, in the context of the current study, is to abstract those design features of interest that can then be idealized in a Computer Aided Design (CAD) environment, to create several designs of varying parameters for evaluation. These are then manufactured and tested to obtain data for final analysis. An alternative approach is to replace or add a computational analysis component to this work, leveraging a coupling between parametric design and analysis, however, this is out of scope in this study. In the following subsections, each of these steps is discussed in more detail.

### 2.2. Digitization

The main aim of digitizing natural honeycomb was to identify features of interest that would serve as design features in the parametric experimental study to follow. Although measurements were made, this was not the primary objective of the study.

#### 2.2.1. Honeybee Comb Specimens

**Figure 5:**
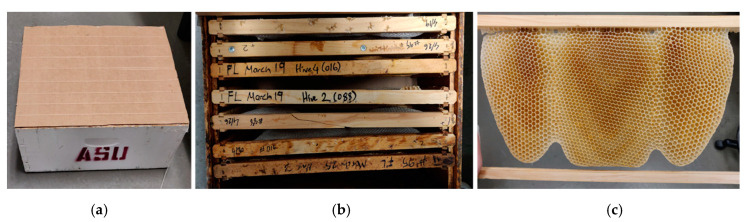
(**a**) Hive box used to encourage *A. mellifera* to construct comb; (**b**) a total of 15 open frames were leveraged; and (**c**) within a few days, the bees had constructed comb.

Honeybee comb was specially obtained for this study, curated at the Bee Lab on the Mesa campus of Arizona State University. The Italian subspecies of the European honeybee, *Apis mellifera ligustica* (hereafter *A. mellifera*) was the species selected for this study. Comb was built in 2 weeks during the period of March–April 2019. Fifteen individual combs from six colonies were created by bees in open frames lacking plastic or wax foundation typically used by beekeepers to more accurately mimic conditions the bees would experience in the wild (Figure 5a,b). This enables a more accurate representation of how these structures are made under natural conditions. The data collected here are on a range of specimens pulled from these honeybee comb—additionally, all reported data are from freshly built comb, since it was collected within 3 weeks of construction. The material composition of comb changes over time as the material of construction transitions from mostly beeswax to a composite of beeswax and silk from spent cocoons [13].

#### 2.2.2. Structured White Light Microscopy

**Figure 6:**
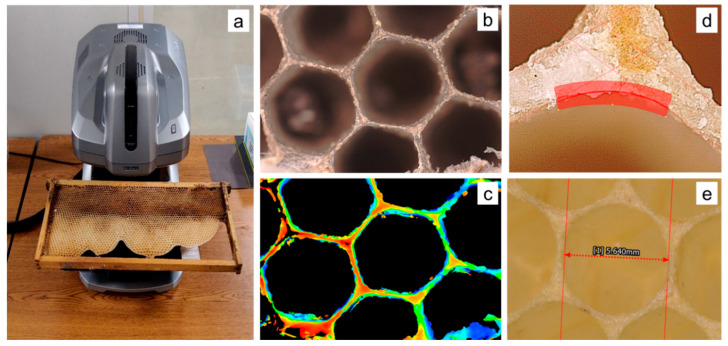
(**a**) Keyence VR-3200 and setup to measure honeybee comb cells, (**b**) an example of optical data on a wasp (*Polistes)* comb, (**c**) height data for the same cells, (**d**) edge detection, and (**e**) an example measurement between two detected edges.

Structured white light microscopy allows for rapid and accurate measurements of geometric features on the order of microns, on relatively large components on the order of several centimeters—each measurement is made for a specific region that fits within the frame of reference, coupled with automatic stitching, and is a rapid data collection technique. For this study, a Keyence VR-3200 (Figure 6a) was used to perform measurements of all two-dimensional geometric features of interest on the outer surface of the honeycomb, in high magnification mode, where the equipment has a supplier-cited 62 micron accuracy. The equipment is accompanied by an analyzing software, which can leverage either optical (Figure 6b) or height (Figure 6c) data for measurements, made using white LED light. In some cases, using the available optical data for edge detection leads to errors in finding an edge or even incorrect detection. Edge detection relies on the user defining where the boundary of specimen is with a box, see Figure 6d. The software then finds an edge within this region and creates a line or arc that best fits the given data. These edges must be defined for measurements to be taken from scan data. Edges are reference points within the data for measurements to be attached to, see Figure 6e for a measurement example. For optical data, edge detection looks at the difference in contrast between pixels in the user-defined box. If the contrast is not significant enough or there is missing data from a scan, edge detection can fail. With the ability to use height data instead, rough surfaces that can cause missing optical data can now be captured and used for edge detection.

**Figure 7:**
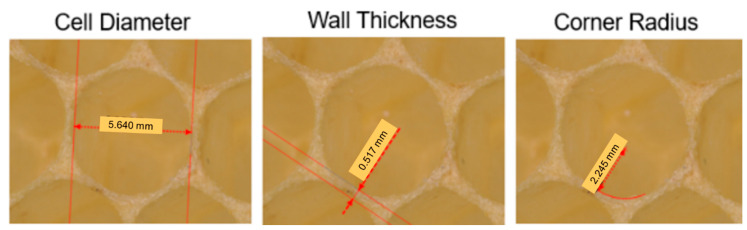
The three parameters measured using the structured white light scanning microscope technique.

A total of three parameters, shown in Figure 7, were measured for this study, all for cells well in the interior of the structure, i.e., at least eight cells removed from the edge. In addition to the corner radius, described previously and one of the metrics of interest, the cell diameter and wall thickness were also measured, to provide normalized datasets, since it is likely that the three parameters are related to each other—a larger cell diameter cell is likely to possess a larger corner radius, with the former driven by the size of the insect that needs to move inside the cell. Cell diameter for a given cell was the average of measurements made from three pairs of opposite walls. Multiple honeybee combs and cells were studied using this measurement technique. For each cell, three cell diameters, six wall thickness, and six corner radii measurements were made.

#### 2.2.3. X-ray Tomography

While optical and scanning electron microscopy (SEM) are used routinely, they only provide information in two dimensions. X-ray microtomography was used to image several aspects of the honeybee comb such as the wall thickness, coping, as well as to make quantitative measurements of corner radii in the cells. An X-ray computed tomography (XCT) scan was performed on a 1 in specimen using a laboratory-scale X-ray microscope (Zeiss Versa 520, Carl Zeiss Microscopy, Pleasanton, CA, USA). An accelerating voltage of 50 kV was used. Briefly, 3201 projections were captured with an exposure time of 6 s per projection. A voxel size of 13.7 µm was achieved using a 0.4× objective lens, a camera binning of 1 and a geometric magnification (ratio of source to detector distance to the source to specimen distance) of 2.5. Following the scan, the dataset was reconstructed using an in-built filtered back-projection algorithm. The as-reconstructed data were filtered using median and anisotropic diffusion filters for denoising and followed-up with an unsharp mask operation to sharpen boundaries. The filtered images were then thresholded using ImageJ to select the pixels belonging to the ligament, which was determined manually. A MATLAB script was written to estimate the thickness of the ligament as a function of depth, with the top of the image being the coping region and bottom being the triple junction.

**Figure 8:**
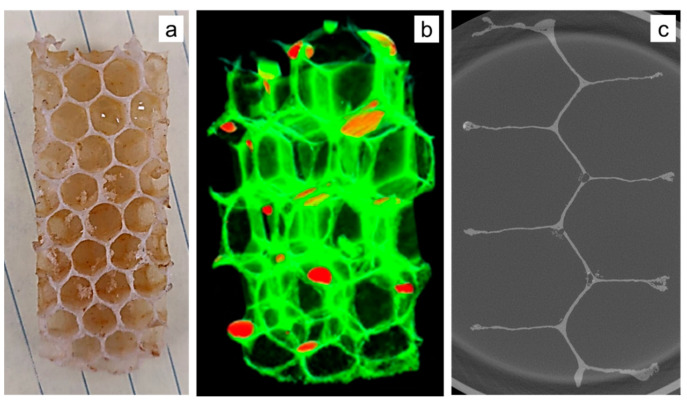
(**a**) Specimen used for X-ray tomography, (**b**) density map showing beeswax and honey, and (**c**) slice image showing coping.

The scanned sample consisted of a structure made of beeswax, with some residual honey. The reconstructed data set was rendered in 3D (Figure 8b) and resliced, such as Figure 8c. Measurements were made along the cell wall using Image software [16]. Coping is clearly visible and formed the basis for closer observation and measurement. Coping is also the reason why the walls look thicker when viewed from above (Figure 8a), as opposed to by the cross-section (Figure 8c).

#### 2.2.4. Silicone Molding

**Figure 9:**
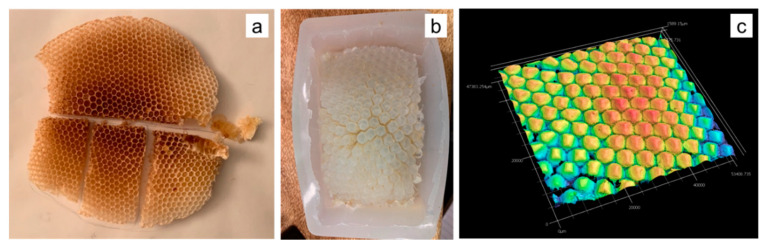
(**a**) Honeybee comb specimen cut up into smaller sections; (**b**) silicone mold created of such a section; and (**c**) white light scanning height data of the mold, showing the nature of the interface.

Silicone molding was used to obtain additional information on the nature of the interface between the honeybee comb cell arrays. A portion was cut out from a comb (Figure 9a) and secured in a soap dish-sized container. Smooth-On Dragon Skin 10 SLOW, a high-performance platinum slow cure pourable silicone rubber was poured over the section due to its ability to capture detail while remaining very strong and stretchy when cured. The material properties of the silicone rubber allow the final cured mold form, shown in Figure 9b, to be flexible without deformation or tearing while examining the interface, surfaces along the crevasses, and other hard to see areas. This mold was then scanned with the previously mentioned structured white light scanning microscope, to obtain height data as shown in Figure 9c. The aim of using this approach was to complement the limited data obtained from X-ray tomography and also have a physical replica of the negative space inside the bee’s honeycomb.

### 2.3. Honeycomb Panel Design

**Figure 10:**
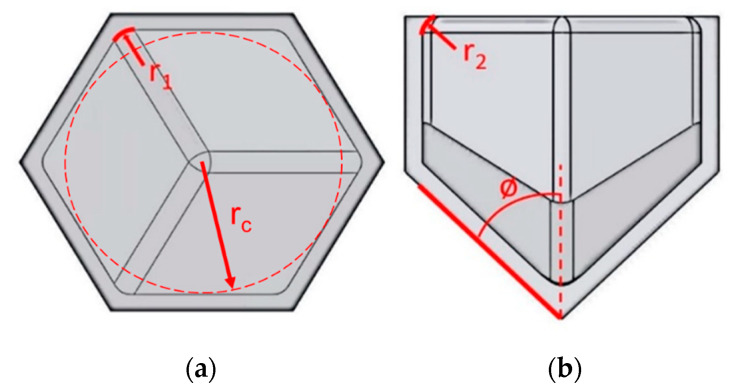
Unit cell showing the three design parameters in this study: (**a**) corner radius *r*_1_ (also shown is *r_c_*, the cell radius, fixed in this study), (**b**) coping radius *r*_2_, and interface angle *ϕ.*

The objective of the design step is to create specimens for testing that reflect a range of values associated with the parameters of interest. All design work for this study was conducted using the SolidWorks 2019 CAD design software [17]. The first step was to create a hexagonal unit cell that embodies the three parameters of interest, as shown in Figure 10. A unit cell with 1 mm thick walls and an 8 mm cell diameter was selected, based on manufacturability assessments to ensure walls were printed with sufficient fidelity and that trapped powder could be adequately removed, and also to ensure that at least 10 cells were present in each direction to minimize size effects [9]. Each of the three parameters was designed following observations of biological comb structure (discussed in Section 3.1). The corner radius (*r*_1_) is shown in Figure 10a and is essentially a fillet radius around the corners of the hexagon. The coping was idealized as a fillet radius (*r*_2_) as well, as shown in Figure 10b; although this does not exactly capture the matchstick like dome at the top of the coping, it accounts for the addition of mass at the very top in a manner that is feasible to model and manufacture to designed intent. Finally, the interface was designed with variable angle *ϕ*, as shown in Figure 10b.

**Figure 11:**
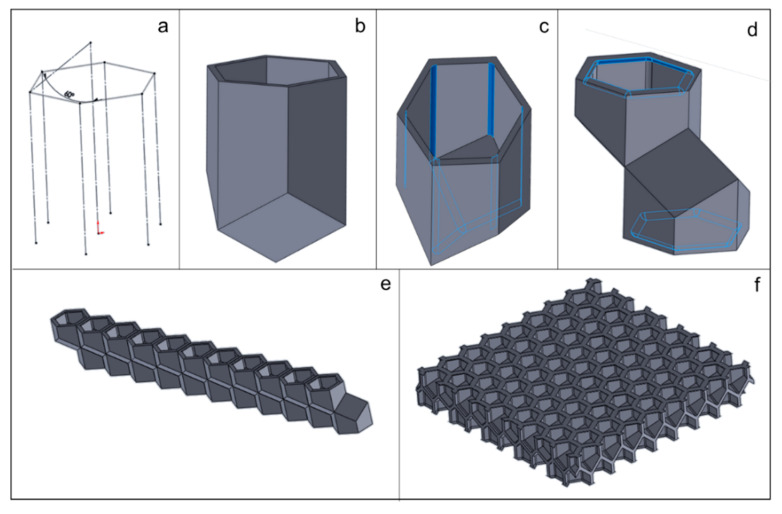
Method used to create specimens for testing: (**a**) construction lines created indicating a hexagonal cell; (**b**) a unit cell created with an interface angle; (**c**) corner fillets created; (**d**) interface created with two opposing cells; and (**e**) array of cells in one direction, (**f**) followed by arraying in the other.

The design steps in the creation of the unit cell, along with its arraying to create a complete, testable specimen, are shown in Figure 11. In addition to the representation of the parameters discussed above, it is crucial to accurately design the relative position of the cells on either side of the interface, as shown in Figure 11d. This two-cell structure is then arrayed in one direction (Figure 11e) and then the other (Figure 11f), to create a honeycomb core specimen that can be manufactured and tested.

**Figure 12:**
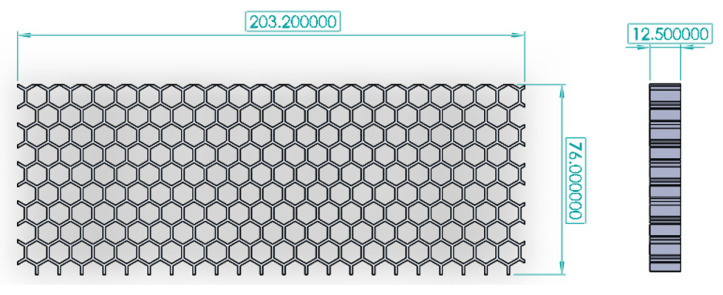
Outer dimensions (in mm) of specimens for the two test conditions, shown here for the 3-point bend specimen. The out-of-plane compression was identical to this specimen with the exception of having a length of 76 mm, matching its width.

The outer dimensions of the specimens, shown in Figure 12, were selected based on guidelines in the MIL-STD-401, a general standard provided for test methods for sandwich core materials [18]. This does result in a fairly thin specimen, especially for the cases with interfaces, but does enable comparisons within the range of specimens studied and also against commercial honeycomb core. Three different categories of specimens were created, for three test conditions: out-of-plane compression, in-plane compression, and three-point bending. For each specimen design, the three design parameters were varied as shown in Table 1, along with a baseline honeycomb core with no corner filleting, coping, or interface (the conventional honeycomb with no interface has a 0° interface angle). This resulted in a total of 64 specimens per test condition or a total of 192 unique designs. A few representative design files are available in the Supplementary Materials.

### 2.4. Additive Manufacturing

**Figure 13:**
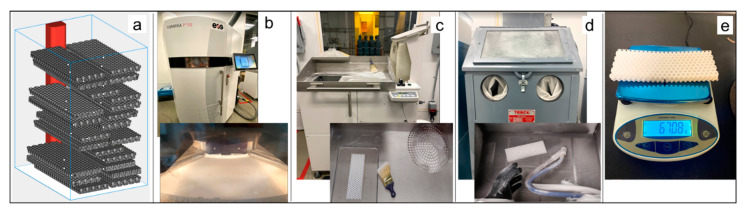
Selective laser sintering (SLS) process flow used in this study: (**a**) part layout in the powder bed, (**b**) fabrication on the EOS Formiga P110 machine, (**c**) part removal from the powder cake, (**d**) trapped powder removal with bead blasting, and (**e**) part weighed prior to testing.

All specimens were manufactured on an EOS Formiga P110 Selective Laser Sintering (SLS) machine. SLS is a laser powder bed fusion technique that is a commercially available AM process for polymers and has the advantages of being able to create fine features without needing support [19]. For this study, polyamide nylon 12 material was used, commercially available from the supplier with the brand name PA2200, which has a tensile modulus and strength of 1650 and 48 MPa, respectively, and an 18% strain at failure, according to the supplier’s datasheet. Commercial SLS parameters for this material, also provided by the supplier, were leveraged. The entire manufacturing process flow is depicted in Figure 13, showing build layout in the build preparation software and postprocessing steps for powder removal. Every part was weighed on an analytical balance prior to testing, to enable normalization by mass for comparison of results.

### 2.5. Mechanical Testing

**Figure 14:**
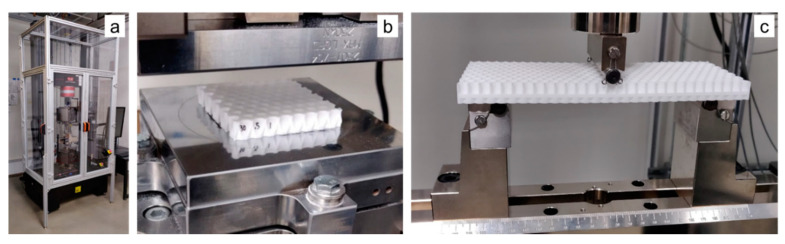
(**a**) Instron 5985 machine used for this study to conduct the following studies: (**b**) out-of-plane compression and (**c**) three-point bending.

Two types of tests were conducted in this study, all on an Instron 5985 electromechanical testing system, shown in Figure 14a. The tests selected for this study were out-of-plane compression (Figure 14b) and three-point bending (Figure 14c). Out-of-plane compression test conditions including strain rates were taken from MIL-STD-401 [18]. Since these materials can be significantly strain rate sensitive even at relatively low strain rates [10], only one strain rate was examined for each test condition. ASTM D790 [20] was referenced for flexural strain rates for three-point bend testing as well as equations for calculating flexural properties. Compression testing was conducted in order to calculate elastic modulus, first maximum stress, and energy absorption. Three-point testing was conducted to calculate maximum load and flexural rigidity.

#### 2.5.1. Out-of-Plane Compression

Out-of-plane compression, as shown in Figure 14b, involves the specimen being laid flat and compressed between platens. A spherical seat was used to conduct these tests, to ensure uniform application of load. A 45 Newton load was applied to the load head to ensure the platens have made complete and uniform contact with one another, after which, the spherical seat was tightened into place. Next, each sample was centered within the compression platens and the test was commenced. An effective strain rate of 2.5 × 10^−3^ s^−1^ was used for the quasi-static compression testing in this study, and the test was conducted to compress the 12.5 mm thick honeycomb by 75%, resulting in a displacement of 9.5 mm, and each test lasted a little over 5 min, consistent with guidelines prescribed in MIL-STD-401 [18].

#### 2.5.2. Three-Point Bending

For three-point bending, a specialized fixture was used, shown in Figure 14c. With the load anvil in the center, roller supports were moved to a distance 152.4 mm apart from one another per MIL-STD-401. Once samples are placed on the test fixture, the cross head is moved down until the load anvil makes contact. The crosshead is then raised in small increments until the load cell displays no load. At this point, the crosshead origin is set, and testing can commence. A flexural strain rate of 0.1 mm/mm/min was used for the three-point bend tests, following recommendations in ASTM D790 [20].

## 3. Results

### 3.1. Honeybee Comb Features

In this section, results of studies made on honeybee comb are reported, i.e., the data and findings coming from a combination of structured white light scanning and X-ray tomography. The focus of this dataset is on the three features of interest (corner radius, coping, and interface), but data on wall thickness and cell size are also reported for purposes of normalization of metrics and comparison to previously published literature. Results were analyzed using linear mixed-effects models (LMM) with the package lme4 [21] in R v3.6.0 [22]. Colony and frame number were included as a nested random effect in each model to control for sampling multiple cells within each frame and colony.

#### 3.1.1. Corner Radius

**Figure 15:**
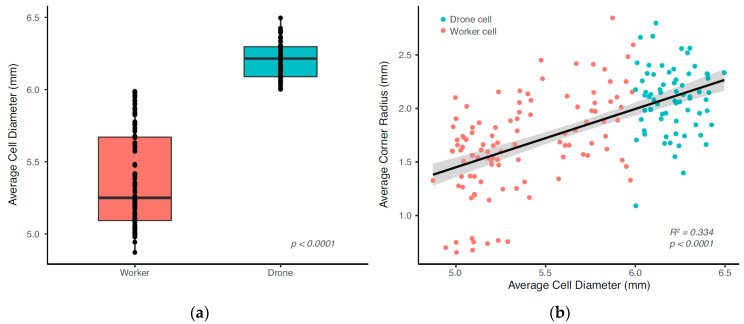
(**a**) Average cell diameter for drone and worker cells from six natural *A. mellifera* comb and (**b**) average corner radius against cell diameter for the same comb.

Data collected from a total of six *A. mellifera* honeybee colonies is compiled here. Bees constructed combs containing both worker and drone cells, which are generally aggregated on specific comb sections. Drone cells are larger than worker cells and can generally be identified by visual inspection. We initially classified cells as either a “worker” or “drone” cell based on visual inspection, and our measurements confirmed that drone cells were indeed larger (LMM, *n* = 179, df = 1, χ^2^ = 194.92, *p* < 0.001), though there was an almost continuous distribution in cell diameters (Figure 15b). The mean values for all drone and worker cells measured was 6.21 mm and 5.42 mm, respectively, which agrees with observations in the literature by Von Frisch [23] and Lee and Winston [24], who report the worker cells as having a cell diameter between 5.2 and 5.4 mm, and the drone cell diameter as between 6.2 and 6.4 mm.

**Figure 16:**
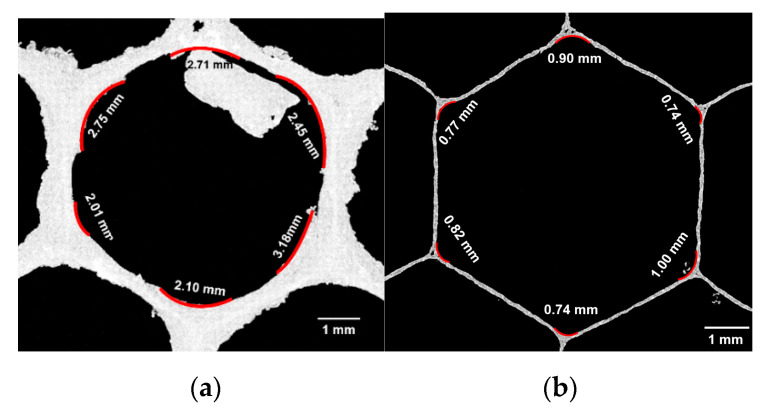
X-ray tomography of a single cell from natural honeycomb, showing (**a**) an average corner radius of 2.53 mm at the top of the coping, and (**b**) a radius of 0.96 mm in the interior of the cell.

Across all cells, there was a significant positive relationship between cell diameter and corner radius (LMM, *n* = 179, df = 1, χ^2^ = 93.31, *p* < 0.001; Figure 15b), suggesting that corner radii are linearly dependent on cell diameter. Corner radii measurements obtained with X-ray tomography data on a single worker cell indicated average corner radii at the coping section measured to be 2.53 ± 0.44 mm (Figure 16a), which is within the range of measurements made with the scanning microscope (Figure 15b). Corner radii at the midpoint between the coping and bottom of each cell to be 0.83 ± 0.11 mm (Figure 16b).

**Figure 17:**
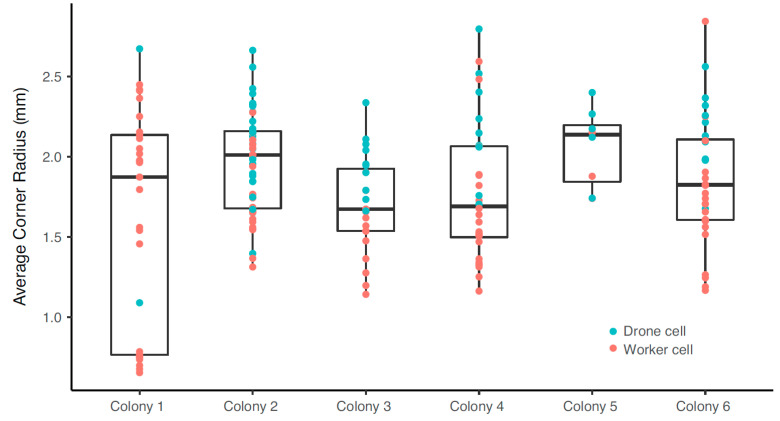
Normalized corner radius for drone and worker cells from six natural *A. mellifera* comb—the mean value is 0.64. A value of 1 represents the point at which the cell has completely transitioned to a circular one.

**Figure 18:**
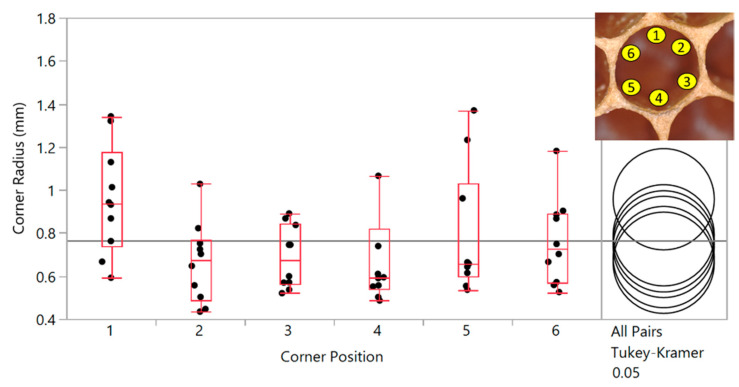
Corner radii by position (shown in inset) shows no statistically significant differences in values (data from one natural *A. mellifera* comb). Variances are equal by Brown–Forsythe test.

To account for the potential dependence of corner radius on cell diameter, and for the difference in cell diameters, the average corner radius was normalized with respect to average cell radius (half the cell diameter—so graphed to enable like comparisons of radii), and shown in Figure 17. The dotted line at the top of the graph corresponds to a complete circle, i.e., when the corner radius equals the cell radius. The mean normalized corner radius for all drone and worker cells combined was found to be 0.64. In other words, the corner radius of a honeycomb cell is 64% of the way to converting the hexagonal cell into a completely circular one, which is remarkable, given the popular idealization of these cells as hexagons with sharp corners. An examination of corner radius by the position of the corner within the cell showed no significant dependence of the value of the radius on which corner was being measured, as shown in Figure 18.

The main finding from this study of corner radius is that the hexagonal cell in honeybee comb has a significant corner radius, which is approximately 64% of a full circle, and suggests that this is a critical design element for the comb’s integrity, given that the formation of the radius requires the allocation of beeswax at the corners. A second finding is that the corner radius scales with cell diameter, suggesting that it is the ratio of the two that is important and should, from a design consideration, be the primary metric of interest.

#### 3.1.2. Coping

In most engineering treatments of honeycomb design, one of the two key parameters is the size of the cell (equivalent to cell diameter, in this study). The other parameter is the thickness of the honeycomb cell walls [5]. Just as the honeycomb cell shape is idealized as a regular hexagon with sharp corners, the thickness of the wall is typically assumed to be constant through the thickness of the honeycomb. Reported thicknesses of the *A. mellifera* comb cell walls in the literature for freshly built comb are given as 73 µm [23] and 88 µm [13]. The latter reference shows how cell walls thicken over time and are thin for a freshly built comb. In both cases, the top coping was removed and measurements were made of the wall underneath it. In this work, the coping itself is of interest, so efforts were made to study the coping geometry, shown previously in Figure 2.

**Figure 19:**
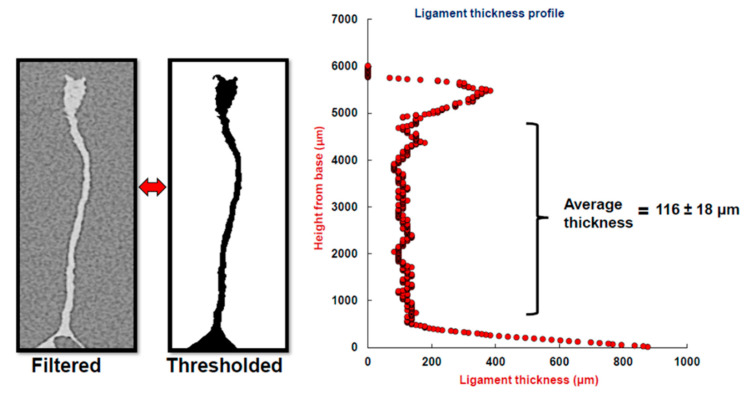
Slice data measurements of cell wall thickness for natural honeycomb from *A. mellifera* using X-ray tomography.

Figure 19 shows a 2D cross-section of a ligament from the base region at the bottom to the coping region on top. The thickness of the wall is fairly uniform from the base region that connects to the interface, right up to the beginning of the bulbous coping region, after which thickness increases rapidly, growing about 5–7-fold at its peak. The coping region is concentrated in a small but significant portion of the wall and, as with the corner radius discussed previously, involves the additional allocation of beeswax material. For seven walls studied, the average wall thickness underneath the coping region was found to be 116 ± 18 µm.

#### 3.1.3. Interface

**Figure 20:**
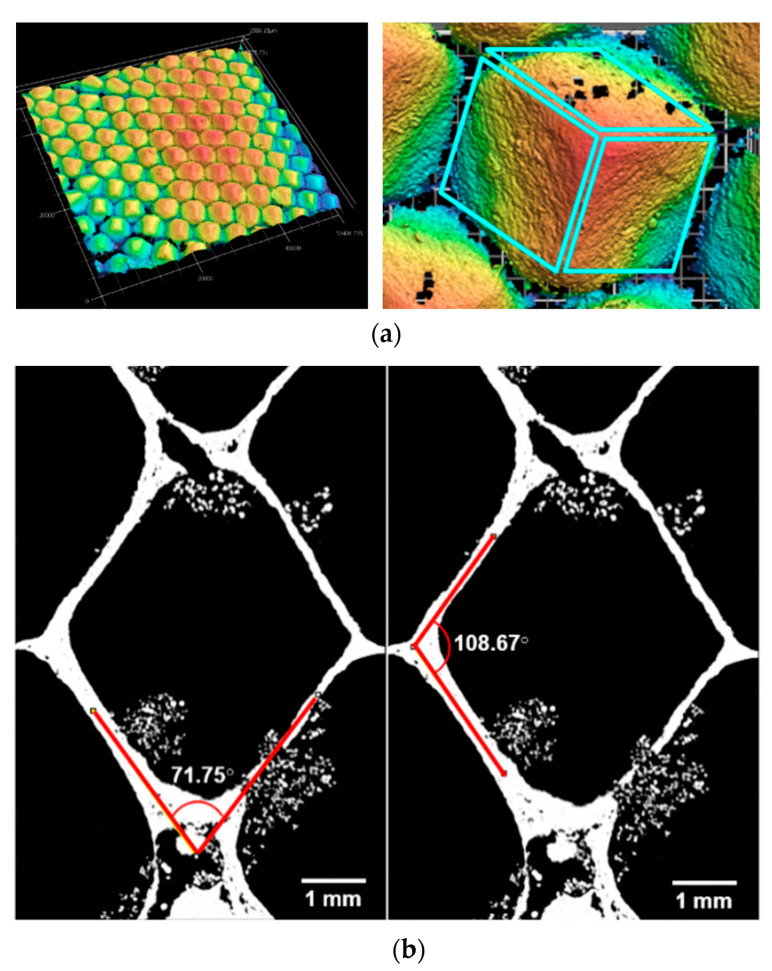
(**a**) Silicone mold showing the three rhombi that form at the bottom of each natural honeycomb cell (**b**) Resliced virtual cross-section showing a rhombus from a honeycomb cell with measurements of its angles. Average corner angle measurements from the three rhombii were found to be 109°58′ and 70°31′.

Unlike most other insect comb, honeybee comb consists of two arrays of cells set back-to-back. As discussed previously, and shown in Figure 20a, each cell interface consists of three rhombi. The question of what angle these rhombi needed to be positioned at for minimizing the quantity of wax needed was first addressed by the Swiss mathematician, Samuel Koenig (1739), who found that the angles (as measured between the edges that make up the rhombi) that fulfilled this condition were 109°24′ and 70°34′ [25]. This agreed well with CT measurements conducted in this work, where average values from measurements on three rhombi in one cell were found to be 109°58′ and 70°31′, as shown for one of these three rhombi in Figure 20b.

### 3.2. Mechanical Testing

From a design standpoint, the specimens that were tested can be viewed as different strategies to allocate additional mass to a baseline honeycomb (with no interface, coping, or corner radius). Of interest in this work is how the additional mass improves *specific* properties, i.e., properties estimated per unit mass and how these compare to the baseline honeycomb, with the lowest mass. As such, the results are presented with an emphasis on properties normalized by mass.

#### 3.2.1. Out-of-Plane Compression

**Figure 21:**
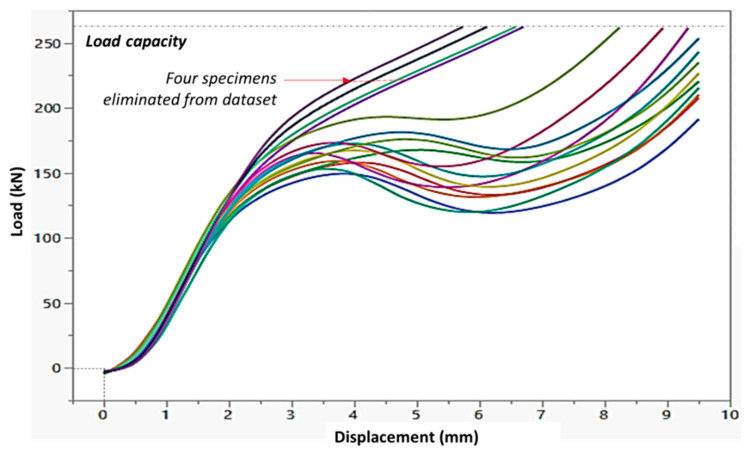
Out-of-plane test output data for manufactured honeycomb in raw load-displacement format showing typical response for specimens tested—four of these specimens exceeded load capacity without any significant data collection and were excluded from subsequent analysis.

Typical out-of-plane compression data obtained for the samples studied is shown in Figure 21 the majority of specimens showed a load-displacement response similar to the effective stress–strain graph shown in Figure 21. Effective stress and strain calculations were made using area and gauge length estimated from the bounding box volume of the specimen, as shown in Figure 12. A MATLAB code was developed to extract the effective modulus, first maximum stress, and energy absorbed (area under the curve). Since all tests were stopped at 0.76 strain, corresponding to a displacement of 9.5 mm, energy calculations are limited to that value. As shown in Figure 21, some specimens did not show a reduction in load and reached the 250 kN load capacity of the machine, causing the test to be stopped at that point. As a result, data from these four specimens are not reported. All four of these specimens that were removed from subsequent analysis had a 30° interface angle, and three of these had 2 mm coping radii.

**Figure 22:**
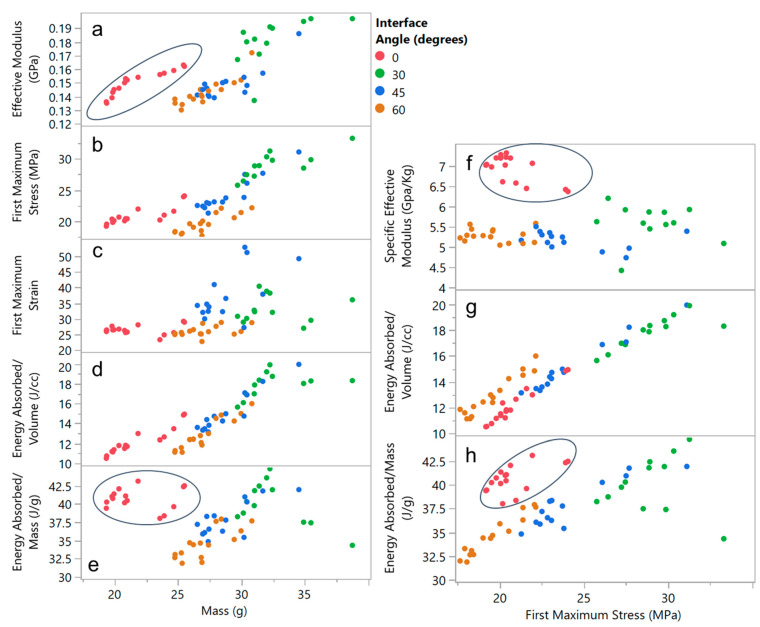
Out-of-plane manufactured honeycomb compression data: (**a**–**e**) metrics of interest graphed against mass and (**f**–**h**) as a function of first maximum stress. Encircled data-points correspond to honeycomb designs without interfaces that deviate from the overall trend.

For the out-of-plane compression test, key metrics of interest are the compressive modulus, the first maximum stress and strain, and the energy absorbed. As shown in Figure 22, all of these metrics generally show an increasing trend with increasing mass. In fact, it is the honeycomb without an interface (i.e., an interface angle of zero) that stands out from the rest, showing higher effective modulus and energy absorption per unit mass, as shown in the encircled regions of Figure 22a. A key design objective for energy absorption is maximizing energy absorbed per unit mass and volume, for a given maximum transmitted stress [26,27]. The graphs in Figure 22b show that the presence of an interface negatively impacts energy absorption per unit mass in this regard. Normalization with regard to mass also enables comparison to other commercial and published honeycomb core data, e.g., the specific energy absorption (SEA) for natural fiber based honeycomb core materials has been shown to be between 0.4 and 1.5 J/g over a relative density range of 0.1–0.4 [28], which is significantly lower than the SEA reported here with additively manufactured nylon 12. Aluminum honeycomb core has been reported as having SEA of between 1 and 5 J/g [29] and independently between 5 and 15 J/g [30]. The nylon 12 SEA values reported here are between 30 and 45 J/g, which suggests that these materials and designs are promising candidates as energy absorbers. These graphs also make it clear that the biggest modulator of mechanical behavior among the parameters and levels studied is the interface angle and that the presence of the interface only serves to reduce the SEA for a given maximum transmitted stress.

**Figure 23:**
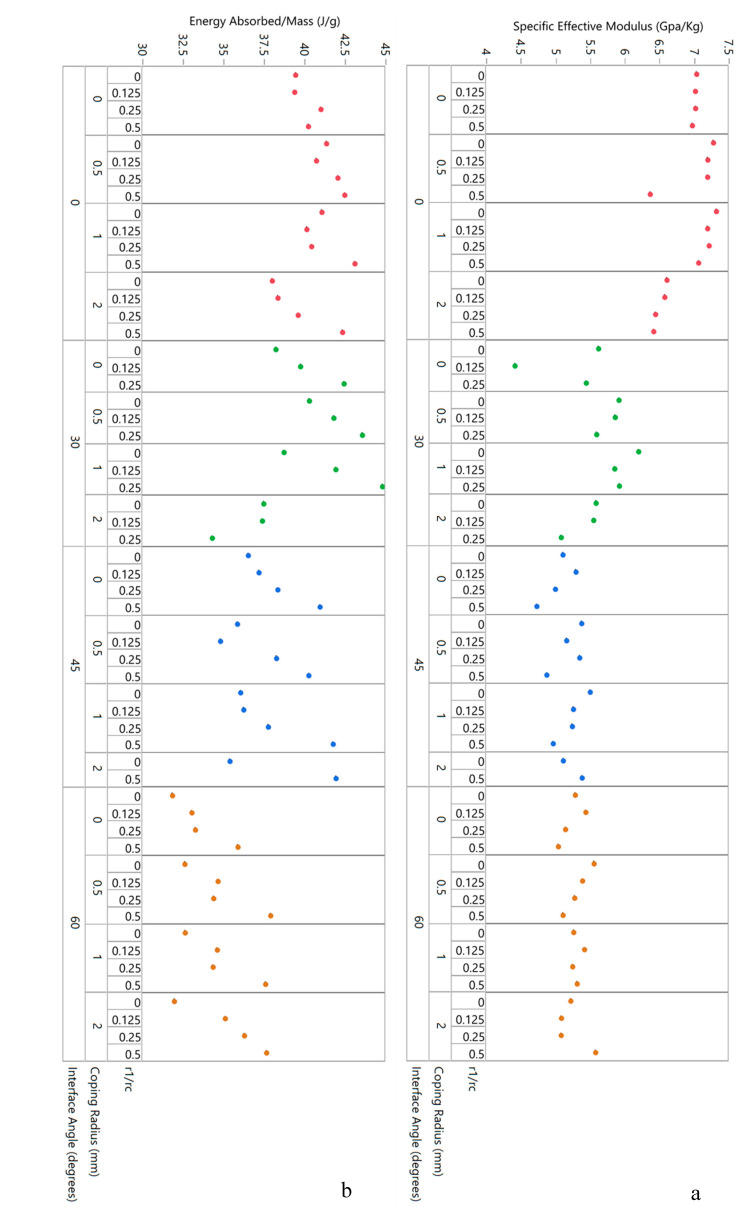
Out-of-plane manufactured honeycomb compression data in variability chart format: (**a**) specific effective modulus and (**b**) energy absorbed per unit mass, both plotted as a function of the three design variables in the study.

Examining the variability charts in Figure 23, it is clear that the specific effective modulus for a given interface angle shows a mild inverse trend with corner radii and is generally unaffected by coping, as shown in Figure 23a. Energy absorption per unit mass, as shown in Figure 23b, is also not only significantly impacted by coping but also shows a stronger and direct relationship to corner radii.

#### 3.2.2. Three-Point Bending

**Figure 24:**
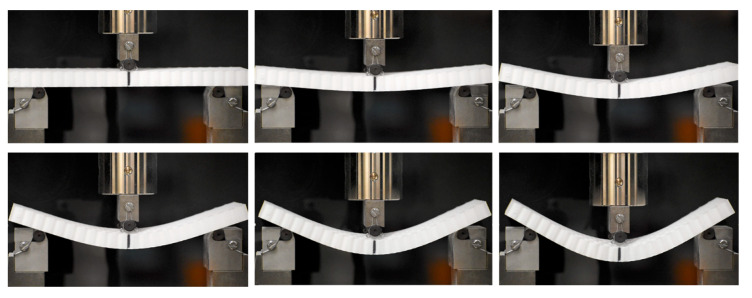
Manufactured honeycomb 3-point bend test progression, with testing carried out till the specimen either had fractured or had gone past its point of peak loading.

Three-point bend testing for the purposes of this study was used to extract two parameters: the flexural strength *σ_f_* and the flexural modulus *E_f_*. The honeycomb core is homogenized as a solid beam under bending, and the two terms of interest are calculated as:

(1) $$\sigma_{f}=\frac{3FL}{2bd^{2}}$$

and

(2) $$E_{f}=\frac{L^{3}m}{4bd^{3}},$$

where *F* is the maximum load measured during the test; *m* is the slope of the initial portion of the load-deflection curve; and *L*, *b,* and *d* are dimensional metrics, representing the span length, the width, and the thickness of the specimen, respectively. A typical bending test progressed as shown in Figure 24 and was carried out till either the specimen had fractured or had gone past its point of peak load.

**Figure 25:**
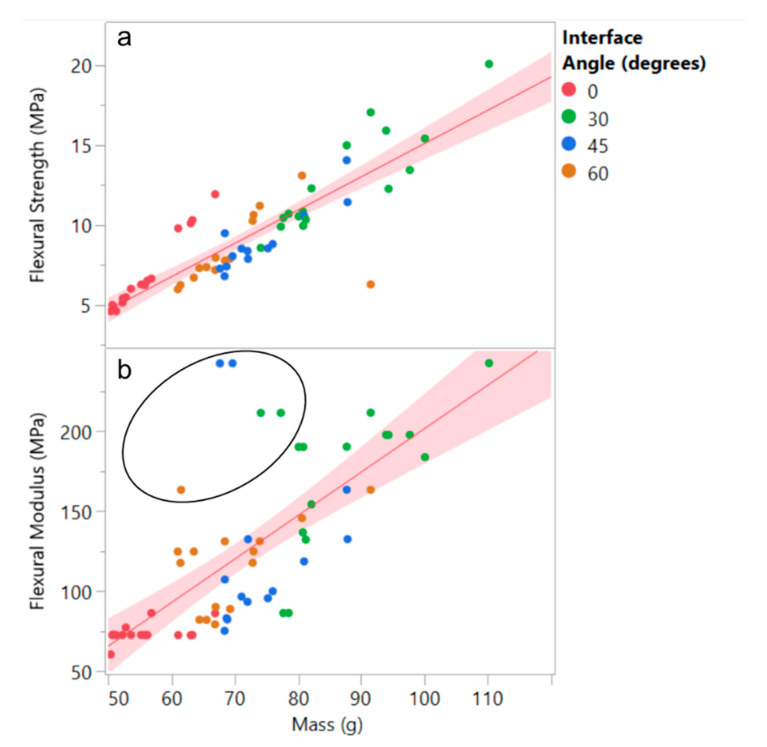
Manufactured honeycomb 3-point bend data showing (**a**) flexural strength and (**b**) flexural modulus graphed against mass. Although strength generally trends with mass, modulus shows deviations (encircled) from it associated with nonzero interface angles.

A total of 62 specimens were tested using this method, each representing a different combination of interface angle, coping radius, and corner radius. The flexural strength and modulus results for these 62 specimens are plotted with respect to measured mass in Figure 25, color coded by the interface angle, with 0° once again representing the baseline honeycomb without any interface. Unsurprisingly, both metrics have a direct relationship with mass of the specimen. Although flexural strength does not reveal any deviations from this relationship (Figure 25a), the flexural modulus does show a few results that significantly improve on the overall trend, as encircled in Figure 25b, all for honeycomb structures with interfaces.

**Figure 26:**
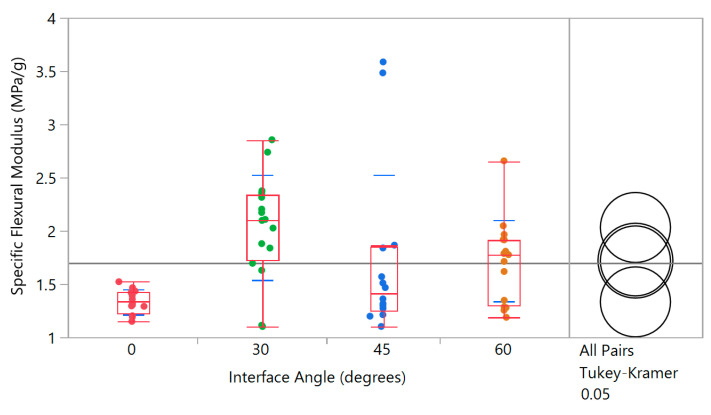
Specific flexural modulus as a function of interface angle (includes results from different coping and corner radii) showing the 30° interface to be statistically better than the baseline 0° (i.e., no interface) for manufactured honeycomb. Variances are equal by Brown–Forsythe test.

To isolate contributions of increasing mass, the flexural modulus can be normalized with respect to mass, to obtain a specific flexural modulus. This specific quantity is plotted against interface angle in Figure 26 and shows that the 30° interface is statistically different from the 0° condition representing no interface. The two results for 45°, while remarkable, can be considered as outliers.

**Figure 27:**
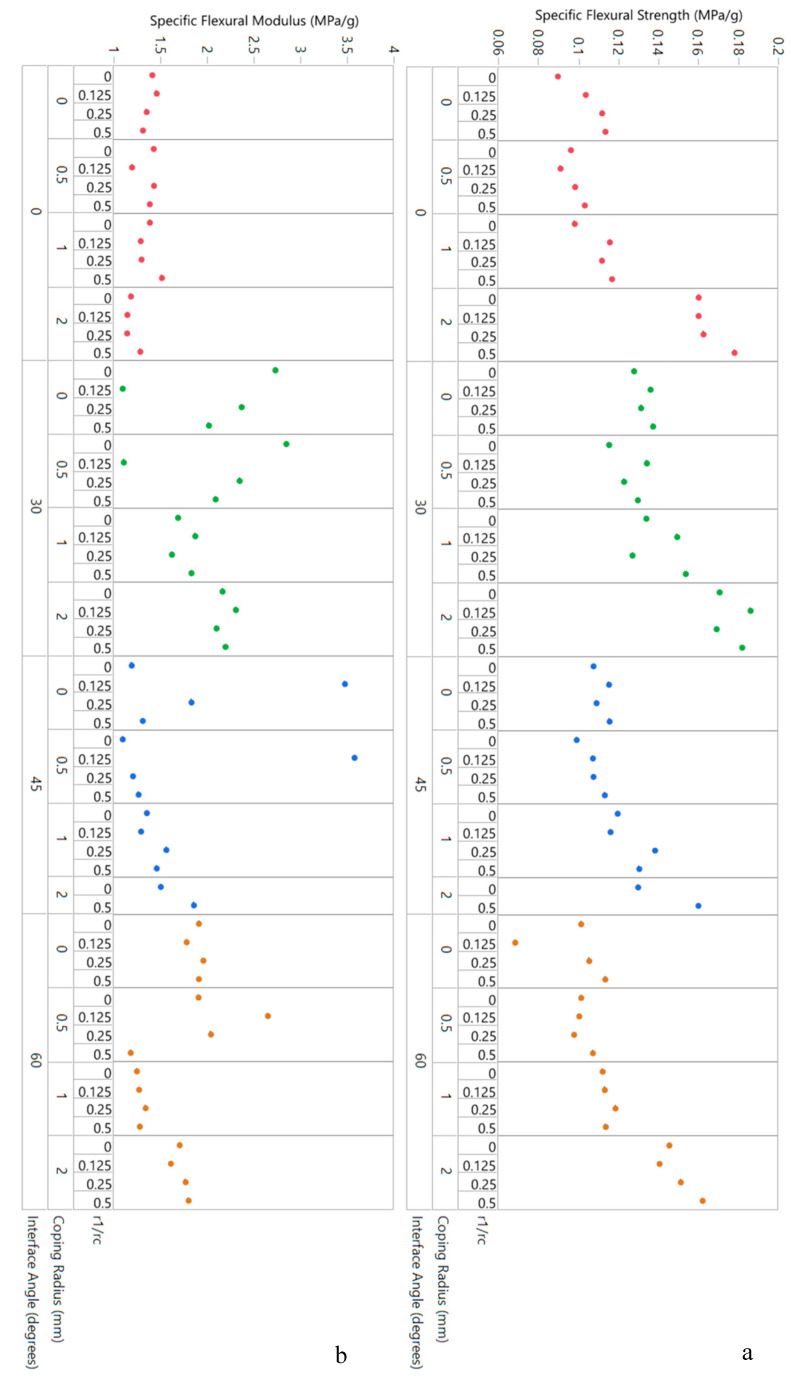
Three-point bending honeycomb data (for manufactured specimens) in variability chart format: (**a**) specific flexural strength and (**b**) specific flexural modulus, both plotted as a function of the three design variables in the study.

**Figure 28:**
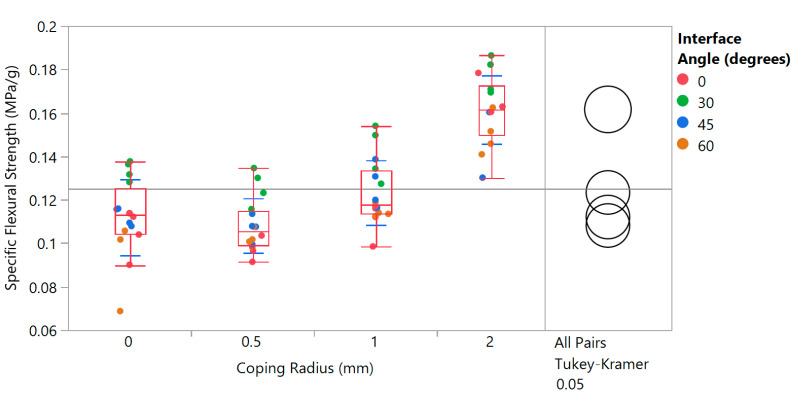
Specific flexural strength of manufactured honeycomb as a function of coping radius (includes results from different interface angles and corner radii) showing an increase with coping radii, with statistically significant differences noticed 1 mm apart. Variances are equal by Brown–Forsythe test.

Finally, the trends in specific flexural strength and specific flexural modulus as a function of the three design variables can be studied in the variability chart in Figure 27. For specific flexural strength, Figure 27a shows that interface angle does not impact results much. For any given interface angle, however, the data suggest that specific flexural strength increases with increasing coping radius—as shown in Figure 28, this effect is quite significant with differences evident every 1 mm. For specific flexural modulus, plotted in Figure 27b, the first remarkable observation is how little the baseline result is affected by any of the design parameters. Here too, it is clear that interface angles do increase specific flexural modulus, with the 30° angle showing the highest values on average.

## 4. Discussion

### 4.1. Material Allocation

All three design parameters studied here, interface angle, coping, and corner radius, are mass-additive. Both in the case of honeybees-constructed comb and engineers design honeycomb core for aerospace applications, to cite a common use of these cores, additional mass is not desirable. In the case of the honeybee, it represents additional calorific expenditure and time spent on sourcing and processing the materials needed for the comb, and for the engineer, it increases the payload of the resulting air- or spaceborne structure, driving up fuel costs. As the experimental results from this paper suggest, however, there are good structural reasons why these additions—even after accounting for the additional mass—may make sense from an engineering standpoint:

- The addition of an interface increases specific flexural modulus (i.e., stiffness under bending) but has little benefit in out-of-plane compression.
- The coping radius strongly influences specific flexural strength—this is perhaps the most remarkable and significant result from the experimental data.
- The corner radius has no significant effect in bending and, actually, is slightly detrimental for out-of-plane compression testing.

There are other test modalities, such as in-plane compression, which have not been evaluated in this study nor has the interaction between the core and the panels that sandwich it been studied in this work. Despite this limitation, these results support further investigation into honeycomb panel cores with interfaces and coping radii.

### 4.2. Structure–Function Relationships

**Figure 29:**
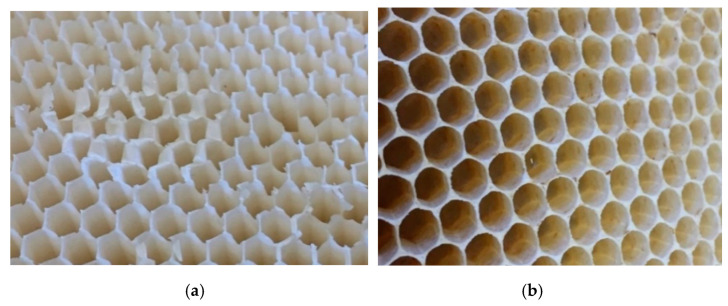
Coping also likely prevents fraying of the thin cell wall for freshly built comb, as suggested by (**a**) an engineered honeycomb structure made from beeswax, which shows fraying of the tops of the cell wall due to handling that is not seen in (**b**) the honeybee’s comb with coping.

The selection of test methods was driven primarily from engineering requirements specified for honeycomb core and less informed by the true functional loading seen by the honeybee’s comb. Additionally, the mechanical behavior of polyamide nylon 12 used in this study is quite different from beeswax. As a result, no strong claims are made on explaining natural honeycomb structure as a result of this study. However, while the primary functional purpose of the honeybee’s comb is storage of brood, honey, and pollen, it nonetheless has to be structurally robust enough to survive one or more seasons in its environment [31]. It may be reasonably argued that the honeybee’s comb design is not optimized for out-of-plane compression, e.g., since that condition does not occur in the honeybee comb environment nor does it experience the large strains in this study. However, this is a common test condition in characterizing honeycomb core and is hence included here in the study. From a structural standpoint, honeybee comb needs to retain integrity under self-weight loading conditions, which is an in-plane loading condition [13]. Additionally, the honeybee’s comb needs to handle wind loads that could impose bending loads on it, on which the 3-point bend test may provide useful insights into. In this context, the corner radius may delay the onset of nonlinear yielding behaviors, and increase bending strength, consistent with what was found experimentally in this work. Finally, the role of coping may also be to prevent fraying of the thin honeycomb walls. Figure 29 demonstrates man-made and natural honeycomb, both made with beeswax, after some months of handling and storage. The engineered honeycomb (Figure 29a) clearly shows signs of walls fraying, while the natural honeycomb does not. This damage tolerance is most likely enabled by the coping, and is another reason why these structures matter.

In summary, this work makes the case for the study of the more nuanced features in honeycomb structure—and by extension to other biological structures, particularly at the interface of bioinspired design and additive manufacturing where such features can be readily realized [32]. Using design, additive manufacturing, and mechanical testing, interfaces and coping were both experimentally demonstrated to be structures that enhance bending performance in particular. Future directions from an engineering standpoint could include a supplementary computational study, additional test modalities, and integration into sandwich panels. Models developed would need to address size effects, both associated with the thickness of the wall, as well as with regard to the number of cells [9,33,34]. From a biological standpoint, there are several structure–function relationships that may be examined here, to identify the selective advantage of the three features discussed here, as well as a comparative study of these features across several comb-building social insect species.

## Figures and Tables

**Table 1:** Experimental plan showing values studied for all three parameters of interest.

Parameter	Baseline	Low	Middle	High
Corner/cell radius ^1^ (*r*_1_*/r_c_*)	0	0.125	0.250	0.500
Coping radius (mm)	0	0.5	1	2
Interface angle (deg.)	0 (no interface)	30	45	60

^1^ Cell radius was fixed at 4 mm.

## Supplementary Materials

STL (CAD) files for representative honeycomb specimens designed for out-of-plane compression and bending are available online at https://www.mdpi.com/2313-7673/5/4/59/s1.
